# Probing the intravascular and interstitial compartments of remodeled myocardium in heart failure patients with preserved and reduced ejection fraction: a CMR study

**DOI:** 10.1186/s12880-018-0301-5

**Published:** 2019-01-05

**Authors:** Pier Giorgio Masci, Anna Giulia Pavon, Gregoire Berchier, Juerg Schwitter

**Affiliations:** 1 Lausanne University Hospital, Cardiovascular Department, and University of Lausanne, Lausanne, Switzerland; 2 Institute of Cardiology, S. Raffaele Hospital, Milan, Italy; 3 Radiology Department, University Hospital Lausanne-CHUV, Lausanne, Switzerland

## Abstract

**Background:**

Recent autopsy studies found microvascular rarefaction in remodeled myocardium of patients who died of heart failure with preserved ejection-fraction (HFpEF). This condition has not been investigated so far by non-invasive methods in patients with HFpEF. The aim was to quantify the intravascular volume (IVV) compartment by CMR in HFpEF patients.

**Methods:**

In two separate CMR examinations, HFpEF patients (*n* = 6; 12 examinations) and post-myocardial infarction patients (post-MI; *n* = 6; 12 examinations) were studied with T_1_-mapping (MOLLI-sequence) before and after IV bolus of 0.03 mmol/Kg of the intravascular contrast-medium (CM) Gadofosveset and 0.2 mmol/Kg of the extravascular CM Gadobutrol yielding IVV and extracellular volume (ECV), respectively. Healthy controls (*n* = 10 with Gadofosveset only, n = 10 with Gadobutrol only) were also studied with the same protocol. IVV and ECV were measured in the basal septum (without ischemic scar in post-MI patients). In post-MI patients, ECV and IVV were also measured in the ischemic scar. Left ventricular (LV) volumes, mass, and ejection-fraction were measured by standard protocol. LV global longitudinal strain (GLS) was calculated by feature tracking on long-axis cine acquisitions.

**Results:**

LV mass to end-diastolic volume ratio and GLS in HFpEF were higher and lower, respectively, than in healthy controls and post-MI patients, whereas the post-MI patients showed lower LV ejection-fraction. Compared to healthy myocardium of controls, IVV in scar was reduced (0.135 ± 0.018 vs 0.109 ± 0.008, respectively, *p* = 0.005), while ECV was increased (0.244 ± 0.037 vs 0.698 ± 0.106, respectively, *p* < 0.001). However, IVV did not differ among HFpEF, post-MI, and healthy controls (0.155 ± 0.033, 0.146 ± 0.038, and 0.135 ± 0.018, respectively, *p* = 0.413), whereas ECV was higher in HFpEF than in post-MI and healthy controls (0.304 ± 0.159, 0.270 ± 0.017, and 0.244 ± 0.037, respectively, *p* = 0.003).

**Conclusions:**

The T_1_-mapping technique combined with an intravascular CM shows potential to measure IVV. In infarct scar with substantially increased ECV, IVV was significantly reduced. Unlike in infarct scar, in remodeled myocardium of HFpEF patients, increased ECV was not accompanied by a reduction of IVV.

## Introduction

Heart failure with preserved ejection fraction (HFpEF) is a common condition with increasing prevalence and high morbidity and mortality [1, 2] HFpEF occurs in association with advanced age, cardiovascular and metabolic comorbidities as well as with a pro-inflammatory state [2]. It is characterized by impaired left ventricular (LV) relaxation and increased diastolic stiffness [2, 3]. Despite intensive research, the myocardial abnormalities subtending HFpEF are incompletely understood. Studies of endomyocardial biopsy or of autopsy of selected HFpEF patients reported myocyte hypertrophy, diffuse interstitial fibrosis and signs of systemic and myocardial inflammation [4, 5]. In this setting microvasculature rarefaction was also observed, and it was inversely correlated to myocardial fibrosis [6, 7]. Accordingly, the detection and quantification of microvascular rarefaction might be of great value for assessing the pathophysiological abnormalities occurring in patients with HFpEF and for establishing its diagnosis. However, the non-invasive measurement of myocardial intravascular compartment in humans is challenging.

Based on these premises, we developed a cardiac magnetic resonance (CMR)-based approach for the quantification of the myocardial intravascular volume (IVV) consisting in the use of an intravascular gadolinium-based contrast medium (CM) combined with the quantitative measurement of myocardial T_1_ by currently available mapping technique [8, 9]. To this end, we administered an intravascular and extravascular CM 1). in healthy controls, 2). in HFpEF patients with LV hypertrophy (LVH) and/or proven interstitial myocardial fibrosis, and 3). in patients with chronic myocardial infarctions (MI). Post-MI patients were included to study the remote non-infarcted myocardium, which typically undergoes remodelling processes with eccentric hypertrophy [10]. In addition, in post-MI patients, we tested the hypothesis that the intravascular CM allows to quantify the reduced IVV, which is a typical feature of post-ischemic infarct scar [11].

## Methods

### Study population

Twenty healthy controls without cardiovascular risk factors, 6 HFpEF patients and 6 patients with previous MI were enrolled into the study. The healthy controls were recruited through local advertising and had no history or symptoms of cardiovascular disease and no cardiovascular risk factors. Patients were recruited from the clinical database of our CMR center after having performed a clinically-indicated CMR examination using an extravascular CM (Gadobutrol, Gadovist®, Bayer Healthcare, Germany). HFpEF patients were selected according to the following criteria: 1) LV-EF > 40% and presence of left ventricular hypertrophy (LVH) and/or T_1_-mapping based extracellular volume fraction (ECV) > 28%, [8]; 2) dyspnea (unless physical activity was compromised by peripheral arterial disease); 3) history of arterial hypertension and ≥ 1 additional cardiovascular risk factor. Exclusion criteria were myocardial ischemia and/or previous MI (by clinical history and on CMR) or known significant valvular heart disease. Post-MI patients were selected on the basis of previous MI (> 12 months) and chronic fibrotic infarct scar on post-contrast CMR. In this subgroup, the scar did not involve the basal interventricular septum. Extensive validation of the T_1_-mapping technique has been performed with regard to standardization of the method [12–14] and in comparison to histopathology when combined with an extracellular Gd-based CM to measure ECV [15]. In this study, we aimed to expand the T_1_ mapping technique to measure the distribution volume of an intravascular CM in order to obtain IVV non-invasively. Accordingly, ECV and IVV were measured by the administration of the extravascular CM gadovist and the intravascular CM gadofosveset (Ablavar®, Lantheus Medical Imaging, US), respectively. In the controls, the two CMs were administered in two separate groups of 10 controls each (to avoid 2 CM administrations in the same subject according to the Local Ethic Committee recommendation). Post-MI and HFpEF patients were studied in a second research-dedicated CMR examination with the administration of the intravascular CM.

The study complied with the Declaration of Helsinki and all examinations were conducted at the Lausanne University Hospital after obtaining the approval from local ethics committee. All study participants provided written informed consent.

### Cardiac magnetic resonance protocol

*In the extravascular CMR protocol*, i.e. in the clinical CMR examination, cine SSFP images were acquired for functional LV evaluation, followed by pre-contrast T_2_-map and T_1_-map acquisitions at the basal short-axis position. At ≥15-min after the i.v. bolus administration of 0.20 mmol/kg of the extracellular CM gadobutrol, the T_1_-map was re-acquired at the same short-axis basal position. Then, late gadolinium enhancement (LGE) imaging was performed on short axis covering the entire LV.

*In the intravascular CMR protocol*, cine SSFP and T_2_-map and native T_1_-maps were acquired as in the extracellular CMR protocol. Then, at 3 min after the i.v. bolus administration of 0.03 mmol/kg of the intravascular CM gadofosveset, the T_1_-map was re-acquired at the same basal short-axis slice position. The 3-min interval was chosen to allow for a homogeneous distribution of the intravascular CM in the subjects’ blood pool, i.e. in the intravascular compartment. This protocol was performed in all patients (6 HFpEF and 6 post-MI patients) and in 10 healthy controls (as described above). In the extravascular and intravascular CMR protocol, hematocrit was measured immediately before CMR.

All studies were performed on a 1.5 Tesla system (Siemens Healthcare, Aera Magnetom, Erlangen, Germany) using dedicated cardiac software, a 32-channel phased array receiver coil, and ECG triggering. The pulse sequence parameters for functional cine SSFP, T_1_- and T_2_-mapping as well as for the LGE imaging are given in Table 1.

**Table 1:** CMR pulse sequence parameters

Tecnique	Sequence	Parameters	Notes on Image Generation
Cine imaging	Breath-hold segmented steady state free precession	flip angle:56°; voxel size: 1,4 × 1,4 × 6,0 TR/TE:37,1/1,19 ms, matrix: 256 × 208; field-of-view: 300 × 240 mm^2^; parlallel imaging acquisition technique: 2; cardiac phases: 25	
T1-mapping	2D Breath-hold MOdified Look-Locker Inversion-Recovery (MOLLI)	Acquisition Sampling Scheme: a) pre-contrast (native): 5 (3)3 b) Post-contrast: 4 (1)3 (1)2 Read-out: SSFP single-shot, trigger delay in mid-diastole, flip angle: 35°, voxel size: 1.5 × 1.5x8mm^3^; parallel acquisition factor: 2, number of inversion pulse 2 and 3 for native and post-contrast	Generation of inline motion corrected pixel-based T1-maps by acquiring a series of images over several heart-beats with shifted inversion delays corrected by RR duration
T2-mapping	2D Breath-hold T2-prepared SSFP sequence	Read-out: single-shot SSFP, trigger delay in mid-diastole, flip angle: 70°, voxel size: 1.9 × 1.9x8mm^3^, matrix size: 192 × 154;; FOV: 360x288mm^2,^ parlallel imaging acquisition technique: 2	Generation of inline motion corrected pixel-based T2-maps by acquiring 3 images with different T2 preparation time (0, 25, 55 ms) with a gap of 2 RR interval within 1 breath-hold
Late gadolinium enhancement	2D breath-hold segmented T1-weighted gradient echo inversion recover with phase sensative reconstraction	Flip angle: 35°; voxel size: 1,3 × 1,3 × 8 mm^3^; matrix size; 256 × 208; TR.TE:848/3,8 ms; FOV: 340 × 275 mm^2^; parallel acquisition factor 2, number of segment: 20 to 30	

### Image analysis

The formula-1 below was applied to calculate the CM distribution volume after administration of the extra-cellular and intravascular CM yielding extracellular volume (ECV) and intravascular volume (IVV), respectively [8].

$$ \mathrm{Distribution}\ \mathrm{volume}=\left(1-\mathrm{hematocrit}\right)\times \frac{\left(1/\mathrm{T}1\mathrm{myo}-\mathrm{post}\right)-\left(1/\mathrm{T}1\mathrm{myo}-\mathrm{pre}\right)}{\left(1/\mathrm{T}1\mathrm{blood}-\mathrm{post}\right)-\left(1/\mathrm{T}1\mathrm{blood}-\mathrm{pre}\right)} $$

T_1-myo-pre_ and T_1-blood-pre_ indicate myocardial and blood native T_1_ values, respectively, and T_1-myo-post_ and T_1-blood-post_ indicate the T_1_ values of myocardium and blood pool after CM administration, respectively.

**Fig. 1:**
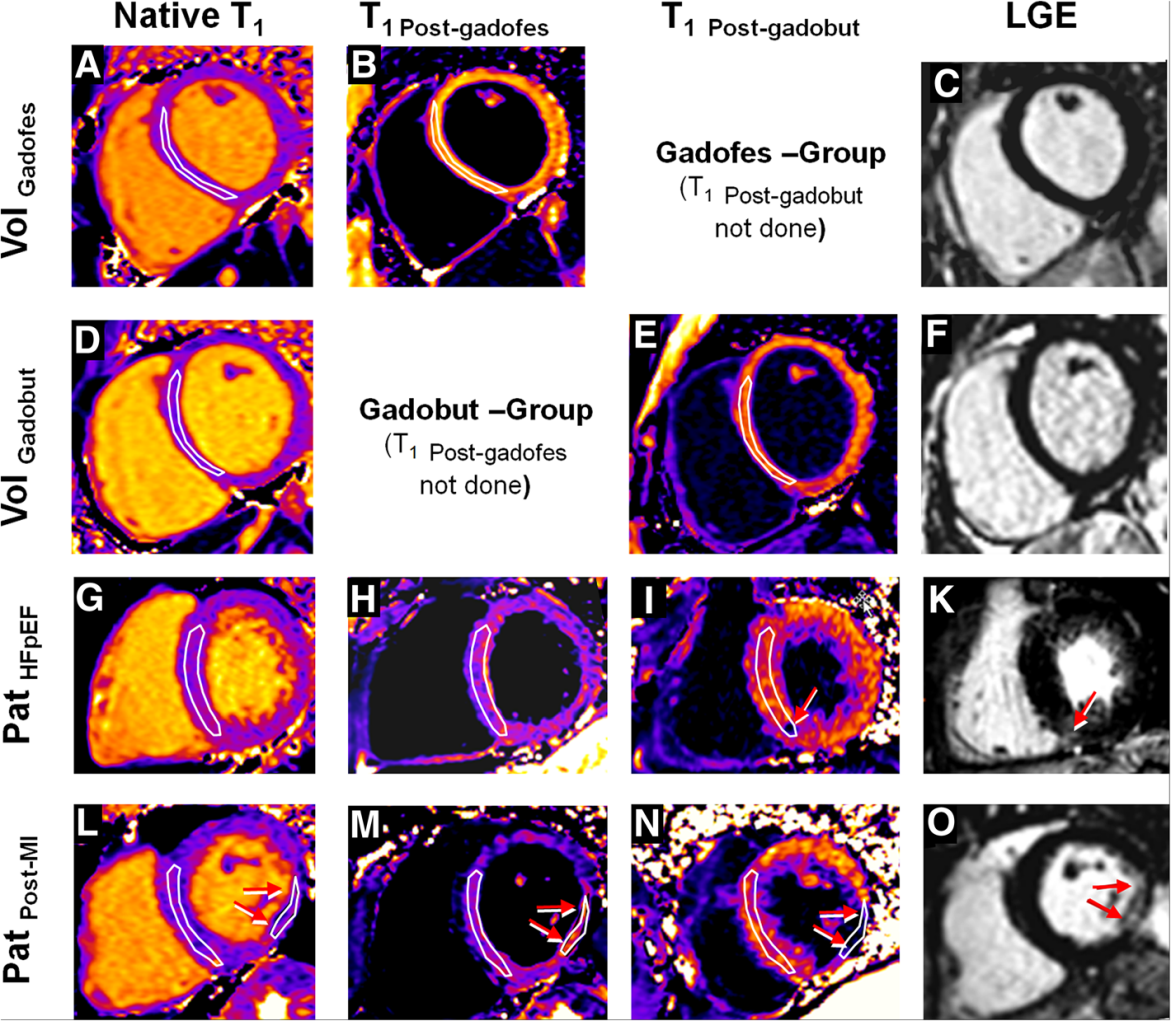
Native and post-contrast T_1_-maps in healthy volunteers, HFpEF, and post-MI patients. First and second rows, basal short-axis native (**a**, **d**), post-gadofosveset (**b**), post-gadobutrol (**e**) T_1_-maps and LGE image (**c**, **f**) in two healthy volunteers. The region-of-interest (ROI) was traced within the basal interventricular septum in all volunteers and patients. Third and fourth rows, in HFpEF and post-MI patients, respectively, show basal short-axis native (**g**, **l**), post-gadofosveset (**h**, **m**), post-gadobutrol (**I**, **n**) T_1_-maps, and LGE images (**k**, **o**). In the HFpEF patient (third row), non-ischemic scars (red arrow in K) was included in the septal ROI for the calculation of IVV and ECV. In post-MI patients (bottom row), a second ROI was traced within the ischemic scar taking as reference the LGE image (**o**, with red arrows indicating the scar)

ECV and IVV were measured 1). in normal myocardium of controls, i.e. in the basal septum; 2). in the basal septum of HFpEF patients, and 3). in the basal septum (=non-infarcted myocardium) and in the infarcted (=LGE positive) myocardium of the post-MI patients (Fig. 1). The interstitial space was calculated as the difference between ECV and IVV.

All studies were saved in DICOM format and analyzed by 1 operator with > 10 years experience in CMR using GTVolume software (GyroTools™, Version 2.2.1, Zurich, Switzerland). For all maps acquired in the basal short-axis, a region of interest (ROI) was manually drawn in the mid-layer of the interventricular septum (segment 2 and 3 according to AHA segmentation). The ROI was drawn by leaving 2 mm from each endocardial side to avoid partial volume contamination with blood pool signal. On T_1_-maps, a second ROI was manually traced in the LV cavity avoiding papillary muscles and trabeculas. These myocardial and blood pool pre-contrast ROIs were then copied to the post-contrast T_1_-maps. Additionally, for post-MI patients (*n* = 6), another ROI was drawn in the infarcted region in the native and corresponding post-contrast T_1_-maps. For a correct identification of the fibrotic scar on the T_1_-maps, a careful side-by-side comparison between T_1_-maps and LGE images was performed. In LGE positive patients the scar mass was quantified as myocardium with a signal intensity exceeding the mean signal intensity of remote myocardium by >6SD using a semi-automatic algorithm [16] and expressed as mass (g) or as percentage of LV mass (%).

### Feature tracking analysis

Two-dimensional Cardiac Performance Analysis Software (TomTec, Munich, Germany) was used to obtain global LV longitudinal strain derived as the mean between longitudinal strain obtained in 2-chamber, 3-chamber, and 4-chamber cine data sets. The mathematical assumptions used and the clinical validation of the feature tracking technology have been previously described [17]. After the upload of the CMR images on a dedicated workstation, the endocardial border of the LV was manually traced in the end-diastolic frame and the software automatically propagated the contour and followed its features throughout the remainder of the cardiac cycle.

### Statistical analysis

Continuous variables are expressed as mean value ± SD and categorical variables are expressed as frequency and (%). Continuous variable differences across healthy controls, HFpEF, and post-MI patients were analyzed by ANOVA, and followed by Bonferroni’s post hoc analyses when required. Categorical variable differences across the groups were assessed by Chi-squared, or by Fisher’s exact test if the expected cell count was < 5. Differences in *p* value < 0.05 were considered statistically significant. All analyses were performed using SSPS version 21 (SPSS, Chicago, Ill-US).

## Results

### Characteristics of healthy controls and patients

The baseline characteristics of the healthy controls and patients are summarized in Table 2. Healthy controls were younger and did not have risk factors as compared to patients. For CMR characteristics, LV volumes did not differ significantly across the groups, whereas LV-EF was consistently lower in patients with previous MI than in healthy controls. In the HFpEF group, LV-EF was preserved with 58 ± 10% [18] with only 1 patient with a LV-EF < 50%, i.e. 48%). LV mass and the ratio between LV mass and LV end-diastolic volume were greater in the HFpEF group than in controls and post-MI patients.

**Table 2:** Baseline characteristics and CMR results

Variable	Volunteer Gadofesveset (*n* = 10)	Volunteer Gadobutrol (*n* = 10)	HFpEF Patients (*n* = 6)	Post-MI Patients (*n* = 6)	Overall *p*-value
Age^a^	35 ± 15	36 ± 11	69 ± 10	58 ± 13	< 0.001
BMI	23 ± 1	24 ± 3	26 ± 3	27 ± 3	0.099
Gender (male) n, %	7 (70)	7 (70)	5 (83)	4 (67)	0.771
Dyspnea (%)^e^	n.a	n.a	4 (67%)	0 (0)	0.014
Symptomtic PAD	n.a	n.a	2 (33%)	0 (0)	
Hypertension n, (%)	n.a	n.a	6 (100)	5 (83)	0.296
Hypercholesterolemia n, (%)	n.a	n.a	4 (67)	3 (50)	0.558
Active Smoking n, (%)	n.a	n.a	4 (67)	3 (50)	0.557
Family for CAD n, (%)	n.a	n.a	1 (17)	1 (17)	NA
Diabetes n (%)	n.a	n.a	2 (33)	0 (0)	0.455
LVEDVI (ml/m2)	87 ± 14	84 ± 10	79 ± 16	95 ± 16	0.247
LVESVI (ml/m2)	33 ± 7	33 ± 5	44 ± 26	52 ± 17	0.064
LVEF (%)^d^	63 ± 6	61 ± 12	58 ± 10	46 ± 11	0.002
LVMI (g/m^2^)^b^	60 ± 11	61 ± 17	85 ± 11	73 ± 16	0.009
LVM/LVEDV^c, b^	0.70 ± 11	0.72 ± 0,17	1.10 ± 0.15	0.77 ± 0.14	< 0.001
Global Longitudinal Strain (%)^b^	−22.78	−21.52	−16.43	−17.07	0.015
Ischemic Scar n, (%)^f^	n.a	n.a	n.a	6 (100)	n.a
Ischemic Scar Extent (% LV mass) ^f^	n.a	n.a	n.a	8.4 ± 2.9	n.a.
Native T_1_ septum (ms) ^a^	1000 ± 19	989 ± 27	1028 ± 31	1029 ± 44	0.029
Native T_1_ blood (ms)	1568 ± 85	1601 ± 110	1607 ± 101	1474 ± 257	0.317
T_2_ septum (ms)	43.8 ± 1.4	45.3 ± 1.8	46.2 ± 1.0	44.7 ± 3.0	0.099
Hct	0.43 ± 0.04	0.043 ± 0.06	0.40 ± 0.06	0.43 ± 0.04	0.726
ECV septum^b^	n.a.	0.244 ± 0.037	0.304 ± 0.0159	0.270 ± 0.017	0.003
IVV septum	0.135 ± 0.018	n.a.	0.155 ± 0.033	0.146 ± 0.038	0.413
Interstitial Space	n.a	n.a	0.149 ± 0.033	0.124 ± 0.044	0.281

^a^Bonferroni post-hoc analysis with *p* < 0.05 between post-MI and HFpEF versus the controls; ^b^Bonferroni post-hoc analysis with *p* < 0.05 between HFpEF versus the controls; ^c^Bonferroni post-hoc analysis with *p* < 0.05 between HFpEF versus post-MI patients; ^d^Bonferroni post-hoc analysis with *p* < 0.05 between post-MI patients versus controls; 1 patient in the HFpEF group with EF < 50% (= 48%). ^e^patients were limited by peripheral arterial disease; ^f^Scar on late gadolinium enhancement. Hct: hematocrit; PAD: peripheral arterial disease

### Distribution volume of intravascular CM in normal myocardium and post-MI scar tissue

**Fig. 2:**
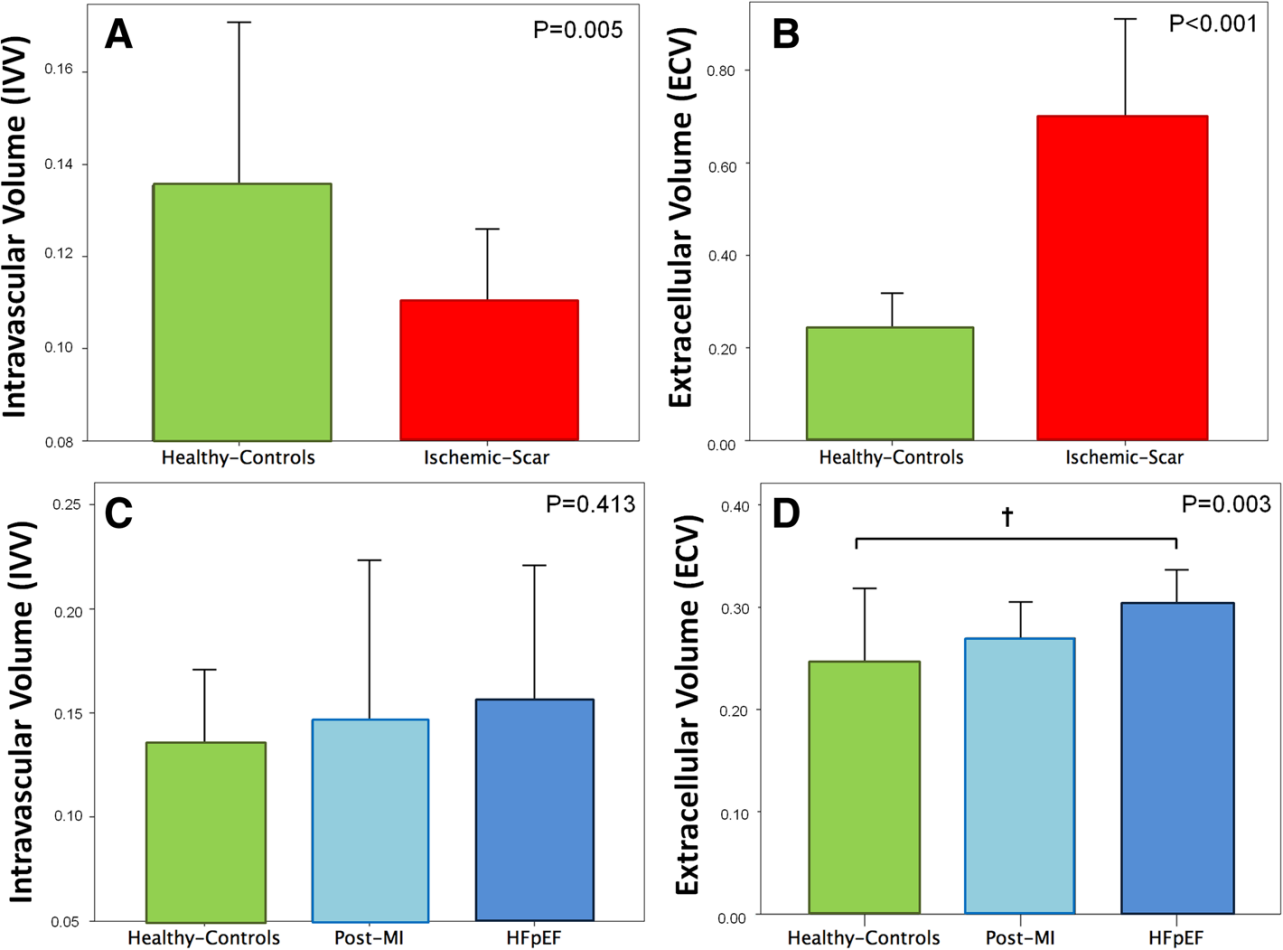
Top Panel: Bars and 95% confidence intervals of intravascular (IVV; **a**) and extracellular (ECV; **b**) volumes in healthy controls (green) and ischemic scar (red). IVV was reduced and ECV was strongly augmented in the ischemic scar as compared to normal myocardium. Bottom Panel: Bars and 95% confidence intervals of IVV (**c**) and ECV (**d**) in healthy (green), post-MI patients (remodeled myocardium not including the scar, light blue) and HFpEF patients (blue). Bonferroni’s post-hoc analysis, † *P* < 0.05

In healthy controls, by applying formula-1, a low distribution volume in the myocardium of 0.135 ± 0.018 was obtained after intravascular CM administration, reflecting the IVV of normal myocardium. In the scar tissue of post-MI patients, IVV was reduced as compared to healthy controls (0.109 ± 0.008 vs 0.135 ± 0.018, *p* = 0.005; (Fig. 2a**)** likely representing the well-known microvascular rarefication in scar tissue, a hallmark of dense fibrotic tissue [11]. The opposite was found for ECV, which was massively elevated in the scar tissue in comparison to normal healthy myocardium (0.698 ± 0.106 vs 0.244 ± 0.037, *p* < 0.001; Fig. 2b).

### Distribution volume of intra- and extravascular CM in non-infarcted myocardium of healthy controls and patients

Native T_1_ value of the myocardium was slightly lower in controls than in HFpEF or post-MI patients, whereas native T_1_ of blood and hematocrit were comparable among groups (Table 2). The IVV of the basal septum did not differ between healthy controls, post-MI and HFpEF patients (Table 2, Fig. 2c. By pooling the IVV data of the basal septum of post-MI and HFpEF patients (*n* = 12), IVV of the basal septum tended to be higher in patients than in healthy controls (0.151 ± 0.034 vs 0.135 ± 0.018, *p* = 0.216). On the other hand, ECV of the basal septum was significantly increased in HFpEF and post-MI patients as compared to the controls (0.301 ± 0.052 vs 0.276 ± 0.023 vs 0.231 ± 0.029, respectively, over-all *p* < 0.001, Fig. 2d).

The T_2_ values of the septum tended to be higher in HFpEF than post-MI patients or controls (Table 2), but were within the upper normal range (48.0 ms) defined as mean plus 2 standard deviations in the pooled data of the healthy controls (*n* = 20, T_2_ = 44.6 ± 1.7 ms).

## Discussion

The main findings of the current study can be summarized as follows. First, in patients with HFpEF, there was no reduction of the myocardial intravascular compartment as derived by T_1_-mapping after gadofosveset administration, while the extracellular compartment was expanded as compared to controls. Second, in the scar tissue of post-MI patients the intravascular compartment was significantly reduced, while the extracellular compartment was massively increased. Third, myocardial edema was not detected in the remodeled myocardium of HFpEF patients as evidenced by the normal T_2_ values.

By assuming that the vascular endothelium is not permeable to albumin under normal conditions, gadofosveset, which binds reversibly to albumin, behaves preferentially as an intravascular CM after intravenous bolus injection. Using T_1_-mapping, we calculated the relaxivity of myocardium and blood pool immediately, i.e. at 3 min, after gadofosveset bolus injection, and these measurements, in conjunction with the hematocrit, were used to derive the myocardial IVV by adopting the previously published formula [9, 11]. The IVV was significantly decreased in the myocardial scar of post-MI patients as compared to normal myocardium of healthy controls, indicating that this approach is able to detect a reduced intravascular space, which is a key feature of dense fibrotic scar [11]. When estimating IVV by the administration of an intravascular CM, it is important to recognize that T_1_-mapping measures the effect of CM on proton relaxation, not reflecting directly the concentration of CM in the intravascular compartment. Although gadofosveset is a prevalently intravascular CM, it affects the extravascular compartment by proton (i.e. water) exchange across the vascular wall [19]. Several studies suggested a slow exchange regime for the intra – extravascular water [19]. Simulations and in-vivo measurements by Donahue and co-workers also demonstrated an increasing overestimation of IVV with increasing intravascular CM dosages (i.e. with an increase in pre-contrast to post-contrast difference of blood relaxivity ΔR_1_). For the slow exchange model, simulations found an overestimation of IVV by approximately 10–30% at a ΔR_1_ of 9, which is matching the ΔR_1_ found in our study (at 3 min after gadofosveset injection) [19]. After IV injection, ~ 70% of circulating gadofosveset binds reversibly to albumin resulting in an increase in relaxivity by 5- to 10-fold (r_1_ = 25 mmol^.^L^-1.^s^− 1^) as compared with the free fraction (r_1_ = 5 mmol^.^L^−^ 1^.^s^− 1^) [20]. The unbound fraction of gadofosveset behaves as an extra-vascular extra-cellular CM, thereby increasing tissue relaxivity in the extravascular space and thus, overestimating IVV. Although we acknowledge that our protocol may lead to an overestimation of IVV by means of either mechanisms mentioned above, it should be kept in mind that overestimation is expected to occur systematically across the diverse study groups and should therefore not affect the trend of the observed pathophysiological abnormalities (e.g., increase or decrease of IVV). Taking into account that the unbound fraction of gadofosveset behaves as an extracellular extravascular CM, the overestimation of IVV is expected to be larger in those pathological conditions with an expanded extracellular compartment (i.e. larger ECV). Nevertheless, our approach showed an important decrease of IVV in the ischemic scar for which we measured a substantial expansion of the extracellular compartment in the order of 70% using an extracellular CM (ECV of 0.698 ± 0.106). Our results are in line with a study by Prech and co-workers [11], who quantified capillary density in autopsy hearts. In that study, in post-MI patients, capillary density was reduced in post-ischemic non-reperfused scar tissue by approximately 40% versus normal. In infarcted myocardium, we found a reduction of IVV of about 25% versus normal, i.e. less than that reported by Prech et al. [11]. This difference matches the expected slight overestimation of 10–30% by our method as discussed above. With this reasoning, it follows that the unbound fraction of gadofosveset is very unlikely to contribute significantly to IVV overestimation in HFpEF patients, who had only slightly increased ECV as compared to healthy volunteers. In summary, we set out a non-invasive CMR approach to estimate IVV by measuring T_1_-maps before and at 3 min after intravascular CM bolus injection, which was demonstrated in ischemic scar versus normal myocardium of healthy controls. Reduced IVV of chronic ischemic scar is well established in the literature, and our approach was capable to correctly gauge this key feature of dense chronic fibrotic scar.

When applying the IVV measurement to non-infarcted myocardium, IVV was not reduced in HFpEF myocardium or in non-infarcted myocardium of post-MI patients. Hence, our results do not confirm some of the previous studies in HFpEF [6, 7]. In particular, in an autopsy cohort of 124 patients who died of HFpEF, Mohammed et al. reported lower coronary microvascular density and more severe fibrosis as compared to control subjects [6]. These authors also found an inverse relationship between microvascular density and fibrosis. However, in their study the HFpEF cohort was somewhat heterogeneous being constituted by 37% of patients with a previous history of MI, and as many as 74% of patients showed LV dilatation and hypertrophy. On the other hand, in our study none of the HFpEF patients had a history of coronary artery disease, evidence of ischemia, or ischemic scar, and none was in advanced stage of heart failure. Conversely, our results are in line with the study by Prech and co-workers [11], who quantified capillary density in autopsy hearts of post-MI patients. In that study, capillary density in remote non-infarcted myocardium was not different from normal myocardium of control hearts.

While slow water exchange was addressed as a potential confounder for measuring IVV in chronic fibrotic post-ischemic scar, additional confounders should be considered when interpreting our IVV results for non-infarcted remodeled myocardium. The concept of using an intravascular CM to quantify IVV is strictly based on the assumption that the intravascular CM does not leak into the interstitial space. In the situation of an inflammatory state of the microcirculation of remodeled myocardium as proposed in the literature [2, 21], leaking intravascular CM would cause overestimation of IVV, in particular, if one considers the higher relaxivity of the albumin-bound fraction of Gadofosveset. To minimize this effect, in the current study T_1_ was measured at 3 min. Post CM injection, as Pedersen et al. [20] showed maximum effect of CM leakage occurring at 30 min post-injection in an animal model. Elevated myocardial native, i.e. pre-contrast, T_1_ values result in elevated IVV according the formula we used (page 5). Puntmann et al. found elevated native T_1_ of viable myocardium of post-MI patients [22] and hypertrophied myocardium [23], which correlated with outcome [22]. In agreement with these studies, we also found elevated native T_1_ values of non-infarcted remodeled myocardium compared to controls (see Table 2), and this elevated native T_1_ may contribute to an increase in IVV. Furthermore, the type of the T_1_ mapping pulse sequence may also influence the calculated T_1_ and thus, distribution volumes. With the MOLLI sequence we used, we were able to measure reduced IVV in scar tissue (i.e. even in tissue with massively expanded extracellular space) and in a recent study, this MOLLI sequence performed best in measuring ECV among a set of different pulse sequences in comparison to histology [24].

While we did not observe differences in IVV between healthy controls and HFpEF patients, ECV was expanded in the later, which is very likely related to an accumulation of extracellular matrix in the cardiac interstitium, since myocardial edema was excluded by normal myocardial T_2_ values. Accordingly, our data on ECV, i.e. on interstitial fibrosis, are in line with other studies, which reported an expanded ECV compared to healthy controls and which correlated well with markers of HFpEF severity [15]. Thus, this study is in line with others in quantifying ECV as a measure of collagen accumulation, while the current non-invasive approach to measure IVV could not detect a rarefaction of the microvasculature of hypertrophied/remodeled myocardium.

### Limitations

The small number of patients included represents an important limitation. This was due to the fact that on August 2016, the production of gadofosveset was halted due to commercial reasons not permitting the recruitment of further patients. Both, ECV and IVV were not validated against endomyocardial biopsy. Rarefaction of the microvasculature in scar tissue is well established in the literature [11]. Therefore, it was deemed adequate to use scar tissue as a reference to demonstrate the feasibility of the proposed method to detect reduced IVV in myocardium. While these preliminary data indicate a potential use to probe IVV and microvascular rarefication, the introduction of new intravascular CM must be awaited to be able to further test this approach and to finally assess its potential clinical usefulness.

## Conclusions

The proposed technique exploiting T_1_-mapping in combination with the administration of an intravascular CM is able to detect a reduction of the intravascular compartment in post-infarction scar tissue. However, our study findings do not support the hypothesis of microcirculatory rarefication as a patho-mechanism in the myocardium of HFpEF patients.

## Abbreviations

CM: Contrast medium
CMR: Cardiovascular magnetic resonance
ECV: Extracellular volume
HFpEF: Hear Failure with preserved ejection fraction
IVV: Intravascular volume
LGE: Late gadolinium enhancement
LV: Left ventricular
